# Relative and absolute within-session reliability of the modified Star Excursion Balance Test in healthy elite athletes

**DOI:** 10.7717/peerj.6999

**Published:** 2019-06-12

**Authors:** Roxana R. Onofrei, Elena Amaricai, Radu Petroman, Oana Suciu

**Affiliations:** 1 Department of Rehabilitation, Physical Medicine and Rheumatology, “Victor Babes” University of Medicine and Pharmacy, Timisoara, Romania; 2 ”Pius Brinzeu” Emergency County Hospital, Timisoara, Romania; 3 ”Louis Turcanu” Emergency Children’s Hospital, Timisoara, Romania

**Keywords:** Athletes, Reliability, mSEBT

## Abstract

**Background:**

The Star Excursion Balance Test (SEBT) is commonly used to assess dynamic postural balance both in clinical practice and research. The aim of our study was to assess the within-session relative and absolute reliability of participants’ performance of the modified SEBT (mSEBT) using a single practice trial in healthy elite athletes who were familiar with the test.

**Methods:**

An intra-session repeated-measures design was used to investigate the relative and absolute reliability of participants’ (healthy athletes partaking in sports at a high-risk of ankle sprain injury) performance of the mSEBT. A total of 122 healthy elite athletes from soccer (*n* = 73), basketball (*n* = 15), and volleyball (*n* = 34) performed one practice trial and three test trials within one session, in three directions (anterior, postero-medial, and postero-lateral), for both legs. Intraclass correlation coefficient (ICC), standard error of measurement (SEM), and smallest detectable change at a 95% confidence were calculated.

**Results:**

We found a good to excellent relative within-session intra-rater reliability between the three trials on specified directions, with an ICC (3,1) from 0.90 to 0.95. SEM and SDC_95_ for normalized and composite scores, for both legs ranged from 0.91 to 2.86, and 2.54 to 7.94, respectively.

**Conclusions:**

In conclusion, we report good to excellent within-session reliability for the mSEBT. Our results confirm that the test can be reliably used with only one practice trial in healthy elite athletes familiar with the test.

## Introduction

The Star Excursion Balance Test (SEBT) is a test used to assess dynamic postural control in clinical practice and research settings (Gribble, Hertel & Plisky, 2012). Previous studies have documented its efficacy in detecting postural balance impairments among individuals with chronic ankle instability (Olmsted et al., 2002; Gribble et al., 2004; Hertel et al., 2006; Gribble, Hertel & Plisky, 2012), anterior (ANT) cruciate ligament injury (Herrington et al., 2009), and patellofemoral pain syndrome (Aminaka & Gribble, 2008). The SEBT has also been used to identify athletes who may be at a heightened risk of lower limb injuries (Plisky et al., 2006; Gribble et al., 2016; Stiffler et al., 2017).

Good reliability is essential so that a test can be confidently used for clinical and scientific purposes. The reliability of the SEBT has been evaluated in many studies, showing good to excellent results, although the protocol, population, and tested directions have varied (Kinzey & Armstrong, 1998; Hertel, Miller & Denegar, 2000; Plisky et al., 2006; Munro & Herrington, 2010; Gribble, Hertel & Plisky, 2012; Van Lieshout et al., 2016).

The SEBT demands subjects to maintain single leg balance while maximally reaching along eight directions with the opposite leg. These tasks require strength, adequate range of motion, proprioception, and neuromuscular control (Earl & Hertel, 2001; Olmsted et al., 2002; Plisky et al., 2006; Hubbard et al., 2007; Hoch, Staton & McKeon, 2011). Due to task redundancy while performing in all eight reach directions, a modified version of the modified SEBT (mSEBT) has been recommended. The mSEBT uses only three directions (ANT, postero-medial (PM), and postero-lateral (PL)) and has proven to be efficient in identifying subjects at risk of lower limb injuries or those with chronic ankle instability (Hertel et al., 2006; Plisky et al., 2006; Hertel, 2008; Gribble, Hertel & Plisky, 2012).

The recommended mSEBT protocol is rather time-consuming due to the number of trials required for achieving performance stability (at least four practice trials and three measurements trials) (Hertel, Miller & Denegar, 2000; Robinson & Gribble, 2008; Munro & Herrington, 2010). Considering its value as a predictor of lower limb injuries in athletes, the mSEBT could be used during pre-season physical assessments. Fewer trials would reduce clinician burden and help to facilitate more efficient testing of large numbers of athletes. Considering that a high proportion of elite athletes are familiar with the mSEBT, it is plausible that the number of practice trials could be reduced during the performance of pre-season physical assessments.

Therefore, the aim of our study was to assess the within-session relative and absolute reliability of the mSEBT using a single practice trial in healthy elite athletes familiar with the test. To our knowledge, no previous studies have been published investigating how many practice trials are needed in subjects accustomed to the mSEBT from previous assessments. We hypothesized that athletes familiar with the test do not need several practice attempts, and, if tested on repeated occasions (successive physical examinations), only one practice trial should be enough.

## Materials and Methods

An intra-session repeated-measures design was used to investigate the relative and absolute reliability of the mSEBT in healthy elite athletes participating in sports with high risk of ankle sprain injuries. All assessments were undertaken by a single investigator. The study has been carried out in accordance with the Declaration of Helsinki and was approved by Institutional Ethics Committee (“Pius Brinzeu” Emergency County Hospital Timisoara—144/22.07.2018).

### Participants

The sample size was calculated based on the methods described by Walter, Eliasziw & Donner (1998). For α = 0.05, β = 0.2, ρ0 = 0.7, ρ1 = 0.8, using three repetitions of each directions, a sample size of at least 80 would be necessary.

Inclusion criteria for this study were: (1) elite athletes aged between 18 and 35 years; (2) athletes that were familiar with the mSEBT from the previous assessments, tested by the same examiner twice during previous pre-season physical assessments, using four practice trials; (3) minimum score for each of the question of the Oslo Sports Trauma Research Center questionnaire—full participation without problems/no training reduction/no performance reduction/no symptoms in the last week (Clarsen et al., 2014); (4) no lower limb injury or surgery for at least 6 months prior to testing; (5) no vestibular disorder; (6) no concussion in the last 2 years.

First and second division soccer, basketball and volleyball athletes were assessed using the mSEBT as a part of the July and August pre-season physical assessments. Participants who met the inclusion criteria and agreed to participate in the study read and signed the informed consent form. A sample of 122 participants was included in the present study.

The following participants’ data were collected: age, gender, height, body mass, body mass index, leg length, sport (Table 1). Leg length was measured in centimeters with participants lying supine, from ANT superior iliac spine to the ipsilateral medial malleolus, with a standard tape measure, on each lower limb.

**Table 1:** Descriptive statistics of the study sample.

Characteristics	Total sample	Soccer players	Basketball players	Volleyball players
Sex				
Men, *n* (%)	88 (72.1)	73 (59.8)	15 (12.3)	
Female, *n* (%)	34 (27.9)			34 (27.9)
Age (years), mean ± SD	23.09 ± 5.13	23.75 ± 5.41	24.4 ± 5.6	21.12 ± 3.7
Weight (kg), mean ± SD	74.6 ± 10.95	74.58 ± 7.65	90.03 ± 10.72	67.85 ± 10.39
Height (cm), mean ± SD	181.9 ± 9.17	179.91 ± 7.24	196.87 ± 9.15	179.57 ± 6.54
BMI (kg/m^2^), mean ± SD	22.47 ± 2.18	23.02 ± 1.77	23.2 ± 1.96	20.98 ± 2.41
Leg length (cm), mean ± SD				
Right leg	93.99 ± 6.48	93.12 ± 4.43	104.67 ± 6.14	91.15 ± 5.81
Left leg	93.97 ± 6.5	93.13 ± 4.47	104.6 ± 6.22	91.12 ± 5.8

### Procedure

We used the mSEBT protocol described by Gribble & Hertel (2003), with three reach directions—ANT, PM, and PL, considered in relation with the stance leg. All testing was done in the morning, when dynamic postural balance performance is better, as advocated by Gribble, Tucker & White (2007). One investigator recorded the maximal reach distance in each direction in centimeters. If the participant removed their hands from the hips, lifted the stance leg from the middle of the grid, touched heavily the ground with the reaching foot, did not touch the line with the reaching foot, or did not bring back the reaching leg to the starting position, the trial was considered invalid and an additional trial was performed (Gribble & Hertel, 2003; Gribble, Hertel & Plisky, 2012).

Participants performed one practice trial and after a 1-min rest, completed three trials in the specified directions on each leg. A 5-s rest between trials was allowed. We used the results of each of these three trials to calculate the normalized mSEBT scores. The normalized scores were calculated for each direction according to the formula: normalized score (%) = (reach distance cm/limb length cm) × 100 (Gribble & Hertel, 2003). The testing order for each trial was the following: right ANT, left ANT, right PM, left PM, right PL, and left PL. The composite mSEBT score was calculated as the average of the three normalized reach direction scores for each leg, for each trial, using the formula: composite score = (normalized ANT + normalized PM + normalized PL)/3.

### Statistical analysis

Statistical analysis was performed using Medcalc version 17.9.7 (MedCalc Software bvba, Ostend, Belgium). Descriptive statistics (frequencies, percentages, means, and standard deviation) were used to describe the study sample. All mSEBT measurements were tested for normality with the Shapiro–Wilk’s test.

Reliability refers to the degree to which a test provides a free-error measurement over repeated trials (Weir, 2005; Riemann & Lininger, 2018). Intraclass correlation coefficient (ICC) with 95% confidence interval was used as a measure of relative reliability. The ICC (3,1), a two-way mixed-effects model with absolute agreement, was calculated to assess intra-rater reliability (Shrout & Fleiss, 1979; Koo & Li, 2016). We considered ICC values less than 0.75 as moderate, values between 0.75 and 0.90 as good, and values greater than 0.90 as excellent (Koo & Li, 2016).

Standard error of measurement (SEM) reports the measurement error in the same units as the test and it is an indicator of absolute reliability. Absolute reliability refers to the precision of the test by estimating the expected error (Riemann & Lininger, 2018). SEM was calculated according to the formula: ${\rm{SEM}} = {\rm{SD pooled}} \times \sqrt {1 - {\rm{ICC}}} $ (Atkinson & Nevill, 1998; Weir, 2005). The measurements are more reliable if the SEM values are smaller. (Atkinson & Nevill, 1998). The smallest detectable change at a 95% confidence interval (SDC_95_) represents the magnitude of real change between measurements necessary to exceed error. Lower values for SDC indicate higher reliability (Hyong & Kim, 2014). We calculated the SDC_95_, using the formula: ${\rm{SD}}{{\rm{C}}_{95}} = 1.96 \times \sqrt 2 \times {\rm{SEM}}$ (Weir, 2005).

The significance level was set at *p* < 0.05 for all tests.

## Results

A total of 122 elite athletes with a mean age 23.09 ± 5.13 years met the inclusion criteria and agreed to participate in the study. Female athletes (27.9%) were members of two elite volleyball teams. The male athletes (72.1%) were members of three elite soccer teams (*n* = 73) and of two elite basketball teams (*n* = 15). Table 1 provides a summary of the descriptive statistics of the study sample.

All mSEBT scores were normally distributed. Table 2 shows the average values of all trials for normalized and composite mSEBT scores.

**Table 2:** Normalized and composite SEBT scores.

Directions	Trial 1	Trial 2	Trial 3
Right leg			
Anterior	80.85 ± 2.85	81.15 ± 2.82	81.59 ± 3.04
Postero-medial	95.53 ± 9.97	96.69 ± 9.82	97.33 ± 9.71
Postero-lateral	102.51 ± 10.26	103.76 ± 10.37	104.66 ± 10.42
Composite	92.96 ± 6.53	93.87 ± 6.55	94.53 ± 6.51
Left leg			
Anterior	78.01 ± 6.44	79.41 ± 6.23	80.12 ± 6.21
Postero-medial	96.48 ± 10.15	98 ± 10.97	99.13 ± 10.94
Postero-lateral	104.37 ± 10.75	105.91 ± 10.75	106.64 ± 10.98
Composite	92.95 ± 7.47	94.44 ± 7.75	95.3 ± 7.79

**Note:**

All values are normalized reach distances (%) (reach distance/leg length × 100).

The relative and absolute reliability indices are shown in Table 3. The ICC values for normalized and composite score showed good to excellent relative reliability.

**Table 3:** Intraclass correlation coefficients, 95% confidence intervals, standard error of measurements and smallest detectable change of the normalized and composite scores.

Direction	ICC (3,1)	95% CI	*p*	SEM	SDC_95_
Right leg					
Anterior	0.90	[0.85–0.93]	<0.001	0.91	2.54
Postero-medial	0.93	[0.91–0.96]	<0.001	2.60	7.21
Postero-lateral	0.94	[0.91–0.96]	<0.001	2.53	7.02
Composite	0.93	[0.91–0.95]	<0.001	1.72	4.78
Left leg					
Anterior	0.93	[0.82–0.96]	<0.001	1.66	4.61
Postero-medial	0.94	[0.89–0.97]	<0.001	2.61	7.26
Postero-lateral	0.93	[0.90–0.96]	<0.001	2.86	7.94
Composite	0.95	[0.92–0.98]	<0.001	1.67	4.64

**Note:**

ICC (3,1), intraclass correlation coefficient; 95% CI, 95% confidence interval; SEM, standard error of measurement; SDC_95_, smallest detectable change. All values, except ICC are normalized reach distances (%) (reach distance/leg length × 100).

The ICC (3,1) values for the right leg ranged from 0.90 to 0.94, with the lowest value for ANT direction (ICC—0.90, with a 95% confidence interval [0.85–0.93], *p* < 0.001) and the greatest value for PL direction (ICC—0.94, with a 95% confidence interval [0.91–0.96], *p* < 0.001). Only in the ANT direction, the lower limit of the 95% confidence interval was smaller than 0.90.

For the left leg, the ICC (3,1) ranged from 0.93 to 0.95, with the greatest value for the composite score (ICC—0.95, with a 95% confidence interval [0.92–0.98], *p* < 0.001). The lower limits of the 95% confidence interval for the ANT and PL direction were smaller than 0.90.

The SEM ranged from 0.91% (ANT) to 2.60% (PM) for the right leg, and from 1.66% (ANT) to 2.86% (PL) for the left leg. The SDC_95_ ranged from 2.54% to 7.21% for the right leg and from 4.61% (ANT) to 7.94% (PL) for the left leg. Lower values for SEM and SDC_95_ were observed for the right leg.

## Discussion

Although the reliability, protocol and clinical implications of the SEBT have been largely analyzed, there are no studies investigating the necessary number of practice trials needed in athletes familiar to the test from previous assessments. Considering its value as a predictor of lower limb injuries in athletes, the mSEBT could be used during pre-season physical assessments. The existing protocol of four practice trials is time-consuming. Since pre-season assessments are often conducted on large teams, fewer practice trials would be useful. The measurement time could be reduced and too the physical burden on athletes.

We examined the relative and absolute within-session intra-rater reliability of the mSEBT using a single practice trial in healthy elite athletes familiar to the test.

Since the SEBT has been mainly used to evaluate postural balance in athletes with ankle joint pathology (acute lateral ankle sprain injuries and chronic ankle instability), we chose to include athletes participating in sports with a high risk of ankle sprains, such as soccer, basketball, and volleyball (Wong & Hong, 2005; Agel et al., 2007; Dvorak et al., 2011; Zuckerman et al., 2018).

### Relative reliability

Using only one practice trial in athletes familiar to the test from previous physical assessments, we observed good to excellent relative within-session intra-rater reliability for the mSEBT for normalized and composite scores, with an ICC (3,1) ranging from 0.90 to 0.95. Our data are comparable with those obtained in previous reliability studies, that used four to six practice trials, on smaller groups of healthy subjects (Kinzey & Armstrong, 1998; Hertel, Miller & Denegar, 2000; Plisky et al., 2006; Munro & Herrington, 2010; Hyong & Kim, 2014; Van Lieshout et al., 2016).

When compared with the results of Hertel, Miller & Denegar (2000), our results showed a slightly higher relative reliability for PM (ICC—0.93 for the right leg, and 0.94 for the left leg) and PL directions (ICC—0.94 for the right leg, and 0.93 for the left leg). In their study, Hertel, Miller & Denegar (2000) used a protocol with six practice trials on a sample of 15 soccer players. In ANT direction, Kinzey & Armstrong (1998) reported slightly lower reliability, in a group of 20 healthy subjects, using five reaching trials.

Our results showed a good to excellent intra-rater reliability using only one practice trial in athletes familiar to the test, while Plisky et al. (2006) found only good intra-tester reliability for ANT, PM, and PL directions, using six practice trials (ICC—0.82–0.87) on a sample of 14 basketball player. Our results based on a larger study sample comprising 122 healthy athletes with different specialization (soccer, basketball, and volleyball) were more optimal than those reported by Plisky et al. (2006).

The relative reliability found in the present study was slightly higher for ANT (right leg ICC—0.9, left leg ICC—0.93), PM (right leg ICC—0.93, left leg ICC—0.94), and PL (right leg ICC—0.94, left leg ICC—0.93) directions than the reliability observed by Munro & Herrington (2010) in a group of 22 healthy volunteers (ICC ranging from 0.84 to 0.92 for the normalized scores of all eight directions of the SEBT, with four practice trials).

The current results were similar with those of Hyong & Kim (2014). They reported high intra-rater reliability for all eight SEBT directions using six practice trials, with an ICC (3,1) ranging between 0.88 and 0.96.

Comparable results were observed by Van Lieshout et al. on a study sample of 55 healthy young adults participating in sports at risk for ankle sprains, using the same three directions as we did in our research. Their results showed good to excellent intra-rater reliability for the composite and separate normalized scores of the SEBT for both legs (ICC—0.87–0.93) (Van Lieshout et al., 2016).

Considering that the values of ICC observed in the current study showed a good to excellent intra-rater relative reliability, our hypothesis that using only one practice trial in athletes familiar to the mSEBT could be considered valid.

### Absolute reliability

The SEM and SDC_95_ provide information about absolute reliability; the lower the values are, the higher the reliability is. With only one practice trial in athletes familiar to the mSEBT we found SEM and SDC_95_ for normalized and composite scores, ranging from 0.91% to 2.86%, and 2.54% to 7.94%, respectively. The measurement error value is also important in order to know within what range a subject’s true score will lie (Munro & Herrington, 2010).

According to the normalized SDC_95_ values, only in athletes familiar to the mSEBT evaluated using one practice trial, clinicians should consider important differences between trials higher than 2.54%, and 4.61% in ANT direction for the right leg, and the left leg, respectively. In PM direction, differences between trials should be considered important if they are higher than 7.21% and 7.26% for the right leg and the left leg, respectively. In PL direction, important differences between trials should be higher than 7.02% and 7.94% for the right leg and left leg, respectively. For the composite score, important differences between trials should be higher than 4.78%, and 4.64% for the composite score for right and left leg, respectively.

Our results were lower than the values found by Hyong & Kim (2014) in their study, using a protocol with six practice trials, (intra-tester SEM and SDC of 2.41–3.30, and 7.16–8.99).

The SEM and SDC_95_ recorded in the present study are lower than the values found by Van Lieshout et al. (2016), except the ANT direction for left leg.

The current SEM and SDC_95_ values for the three directions were lower for the right leg than the values obtained for the left leg, showing a more stable performance in maintaining balance on the right leg.

In contrast to our results, Van Lieshout et al. (2016) found systematic lower SDC values for the left leg, probably because of the protocol and learning-effects. Their protocol started always with the right leg as the stance leg. In order to eliminate this learning-effect we used the following testing order: right ANT, left ANT, right PM, left PM, right PL, and left PL.

Whereas previous reliability studies have used at least four practice trials on smaller groups of healthy subjects, our data were similar or even superior to those previous studies. The reliability values found in our study on a larger sample have significant implications in sports settings. Using only one practice trial in athletes familiar to the test will reduce the physical assessment time, the burden on athletes and on tester, especially in the pre-season screening of teams with high number of sportsmen.

Our study has some limitations. One limitation is represented by the fact that the sample was largely male. Soccer, basketball, and volleyball athletes were tested, so the results could not be extended to other sports. The within-session evaluations and fatigue could influence the accuracy of the results. The SEM and SDC_95_ were calculated based on measurements taken from healthy, uninjured young elite athletes and are limited to those who had previous experience with the mSEBT. The results of our study open ways for future work directed at the screening of athletes at risk of lower limb injuries.

## Conclusions

We report good to excellent within-session reliability for mSEBT, using a single practice trial protocol in healthy soccer, basketball, and volleyball athletes familiar to the test from previous assessments. This protocol will reduce the examination time, burden on athletes and tester, without affecting the testing results. Clinicians involved in extended teams’ examinations will benefit from these results taking into consideration the time efficient pre-season screening.

## Supplemental Information

10.7717/peerj.6999/supp-1 Supplemental Information 1 Descriptive characteristics and SEBT scores.

## Additional Information and Declarations

### Data Availability

The following information was supplied regarding data availability: The raw data are available as a Supplemental File.
