# Supplementary material for: Relative and absolute within-session reliability of the modified Star Excursion Balance Test in healthy elite athletes
Source: PeerJ. 2019 Jun 12;7:e6999. doi: 10.7717/peerj.6999 (PMC6571006; doi:10.7717/peerj.6999)
Supplement: Supplemental Information 1 [file peerj-07-6999-s001.docx]

| ID | Sex  (1-male, 2-female) | Age (years) | Weight (kg) | Height (cm) | Sport  (1-soccer, 2-basketball, 3-volleyball) | Right leg length  (cm) | Left leg length (cm) | Right leg | | | | | | | | | Left leg | | | | | | | | |
| --- | --- | --- | --- | --- | --- | --- | --- | --- | --- | --- | --- | --- | --- | --- | --- | --- | --- | --- | --- | --- | --- | --- | --- | --- | --- |
|  |  |  |  |  |  |  |  | Anterior (cm) | | | Postero-medial (cm) | | | Postero-lateral (cm) | | | Anterior | | | Postero-medial | | | Postero-lateral | | |
|  |  |  |  |  |  |  |  | Trial 1 | Trial 2 | Trial 3 | Trial 1 | Trial 2 | Trial 3 | Trial 1 | Trial 2 | Trial 3 | Trial 1 | Trial 2 | Trial 3 | Trial 1 | Trial 2 | Trial 3 | Trial 1 | Trial 2 | Trial 3 |
|  | 1 | 20 | 75 | 187 | 1 | 96 | 96 | 78 | 78 | 80 | 88 | 86 | 89 | 86 | 88 | 89 | 76 | 74 | 75 | 88 | 90 | 92 | 86 | 85 | 86 |
|  | 1 | 19 | 76 | 171 | 1 | 85 | 85 | 75 | 75 | 75 | 100 | 98 | 100 | 108 | 106 | 105 | 76 | 76 | 77 | 92 | 94 | 96 | 97 | 100 | 100 |
|  | 1 | 30 | 65 | 168 | 1 | 83 | 83 | 68 | 68 | 68 | 84 | 85 | 85 | 91 | 94 | 90 | 61 | 61 | 61 | 90 | 92 | 94 | 91 | 93 | 90 |
|  | 1 | 30 | 95 | 195 | 1 | 103 | 104 | 84 | 84 | 85 | 100 | 98 | 100 | 103 | 102 | 105 | 70 | 70 | 70 | 91 | 90 | 93 | 105 | 105 | 105 |
|  | 1 | 31 | 80 | 181 | 1 | 93 | 93 | 73 | 74 | 75 | 100 | 100 | 100 | 108 | 105 | 111 | 70 | 75 | 75 | 100 | 105 | 110 | 108 | 110 | 110 |
|  | 1 | 19 | 65 | 171 | 1 | 88 | 88 | 70 | 70 | 72 | 99 | 98 | 101 | 100 | 105 | 110 | 70 | 69 | 65 | 95 | 97 | 100 | 110 | 108 | 110 |
|  | 1 | 25 | 85 | 188 | 1 | 97 | 97 | 80 | 80 | 80 | 90 | 92 | 95 | 100 | 110 | 110 | 78 | 79 | 80 | 99 | 105 | 102 | 110 | 110 | 110 |
|  | 1 | 32 | 73 | 177 | 1 | 91 | 91 | 70 | 71 | 72 | 88 | 91 | 92 | 110 | 104 | 110 | 71 | 71 | 72 | 98 | 102 | 105 | 110 | 109 | 110 |
|  | 1 | 36 | 84 | 188 | 1 | 96 | 96 | 78 | 80 | 80 | 90 | 88 | 91 | 91 | 91 | 93 | 67 | 68 | 68 | 87 | 89 | 90 | 98 | 100 | 99 |
|  | 1 | 24 | 84 | 186 | 1 | 95 | 95 | 74 | 74 | 75 | 75 | 84 | 85 | 80 | 80 | 81 | 69 | 70 | 71 | 80 | 82 | 83 | 82 | 84 | 85 |
|  | 1 | 27 | 76 | 180 | 1 | 90 | 90 | 68 | 70 | 68 | 82 | 85 | 85 | 84 | 84 | 85 | 68 | 70 | 68 | 84 | 83 | 85 | 88 | 87 | 88 |
|  | 1 | 30 | 86 | 190 | 1 | 100 | 100 | 85 | 86 | 86 | 87 | 90 | 90 | 85 | 90 | 89 | 74 | 76 | 75 | 90 | 92 | 89 | 90 | 92 | 93 |
|  | 1 | 32 | 83 | 183 | 1 | 96 | 96 | 82 | 81 | 82 | 94 | 91 | 90 | 98 | 100 | 100 | 87 | 86 | 85 | 90 | 92 | 87 | 96 | 97 | 99 |
|  | 1 | 20 | 69 | 181 | 1 | 95 | 94 | 80 | 81 | 82 | 75 | 70 | 70 | 75 | 75 | 76 | 65 | 67 | 67 | 70 | 67 | 68 | 80 | 78 | 78 |
|  | 1 | 19 | 61 | 171 | 1 | 90 | 91 | 68 | 68 | 68 | 71 | 70 | 70 | 79 | 81 | 81 | 60 | 63 | 62 | 72 | 70 | 71 | 84 | 85 | 84 |
|  | 1 | 19 | 86 | 186 | 1 | 96 | 96 | 78 | 77 | 77 | 90 | 91 | 90 | 90 | 90 | 92 | 68 | 70 | 70 | 88 | 90 | 91 | 100 | 100 | 97 |
|  | 1 | 36 | 84 | 176 | 1 | 90 | 90 | 72 | 72 | 73 | 97 | 98 | 98 | 102 | 105 | 105 | 63 | 64 | 65 | 97 | 99 | 100 | 102 | 103 | 100 |
|  | 1 | 28 | 90 | 191 | 1 | 102 | 102 | 84 | 83 | 84 | 83 | 85 | 85 | 96 | 95 | 100 | 72 | 70 | 72 | 85 | 88 | 88 | 95 | 95 | 95 |
|  | 1 | 19 | 81 | 190 | 1 | 89 | 89 | 70 | 70 | 67 | 85 | 85 | 88 | 88 | 86 | 90 | 65 | 68 | 68 | 86 | 87 | 90 | 89 | 90 | 90 |
|  | 1 | 19 | 71 | 171 | 1 | 92 | 92 | 72 | 71 | 72 | 84 | 83 | 85 | 84 | 90 | 88 | 62 | 62 | 61 | 82 | 80 | 80 | 85 | 85 | 87 |
|  | 1 | 22 | 75 | 183 | 1 | 92 | 92 | 70 | 72 | 72 | 87 | 90 | 91 | 85 | 90 | 90 | 67 | 69 | 70 | 90 | 94 | 95 | 91 | 94 | 92 |
|  | 1 | 29 | 70 | 176 | 1 | 89 | 89 | 70 | 70 | 70 | 87 | 90 | 91 | 90 | 95 | 95 | 69 | 71 | 70 | 87 | 88 | 90 | 94 | 93 | 95 |
|  | 1 | 29 | 76 | 180 | 1 | 90 | 90 | 72 | 74 | 74 | 90 | 92 | 94 | 104 | 105 | 110 | 70 | 72 | 73 | 99 | 101 | 100 | 104 | 100 | 105 |
|  | 1 | 31 | 70 | 181 | 1 | 93 | 93 | 70 | 72 | 72 | 90 | 90 | 81 | 90 | 90 | 87 | 67 | 68 | 70 | 79 | 83 | 82 | 81 | 84 | 82 |
|  | 1 | 27 | 78 | 178 | 1 | 88 | 88 | 70 | 71 | 72 | 80 | 90 | 88 | 88 | 90 | 90 | 61 | 59 | 60 | 94 | 85 | 96 | 88 | 90 | 90 |
|  | 1 | 18 | 79 | 171 | 1 | 96 | 96 | 74 | 75 | 75 | 99 | 98 | 97 | 97 | 99 | 98 | 67 | 68 | 70 | 95 | 94 | 97 | 98 | 100 | 99 |
|  | 1 | 21 | 80 | 182 | 1 | 94 | 93 | 70 | 70 | 72 | 70 | 68 | 70 | 78 | 78 | 80 | 68 | 70 | 69 | 69 | 68 | 72 | 75 | 77 | 77 |
|  | 1 | 20 | 75 | 190 | 1 | 96 | 96 | 75 | 75 | 75 | 87 | 90 | 91 | 95 | 94 | 95 | 73 | 75 | 75 | 86 | 88 | 90 | 100 | 100 | 98 |
|  | 1 | 33 | 70 | 172 | 1 | 93 | 93 | 70 | 72 | 70 | 80 | 80 | 82 | 85 | 83 | 80 | 68 | 69 | 70 | 82 | 84 | 81 | 89 | 90 | 90 |
|  | 1 | 18 | 72 | 172 | 1 | 90 | 90 | 72 | 74 | 74 | 68 | 70 | 73 | 70 | 72 | 72 | 68 | 70 | 71 | 69 | 70 | 68 | 70 | 75 | 74 |
|  | 1 | 25 | 71 | 182 | 1 | 96 | 95 | 76 | 76 | 77 | 82 | 80 | 90 | 100 | 100 | 105 | 73 | 74 | 75 | 88 | 87 | 90 | 98 | 100 | 101 |
|  | 1 | 18 | 82 | 192 | 1 | 100 | 101 | 80 | 81 | 82 | 90 | 90 | 93 | 102 | 100 | 105 | 79 | 82 | 83 | 90 | 88 | 91 | 93 | 92 | 95 |
|  | 1 | 21 | 75 | 175 | 1 | 90 | 90 | 72 | 73 | 73 | 70 | 80 | 78 | 87 | 88 | 90 | 73 | 75 | 76 | 82 | 84 | 83 | 87 | 88 | 90 |
|  | 1 | 28 | 73 | 175 | 1 | 91 | 92 | 73 | 74 | 75 | 80 | 80 | 82 | 83 | 85 | 85 | 73 | 74 | 75 | 72 | 74 | 74 | 80 | 80 | 82 |
|  | 1 | 25 | 81 | 184 | 1 | 99 | 99 | 82 | 83 | 82 | 116 | 118 | 115 | 116 | 117 | 119 | 82 | 81 | 83 | 115 | 118 | 120 | 118 | 122 | 118 |
|  | 1 | 23 | 68 | 179 | 1 | 95 | 95 | 78 | 80 | 80 | 68 | 70 | 70 | 80 | 80 | 84 | 80 | 79 | 78 | 70 | 72 | 75 | 78 | 79 | 80 |
|  | 1 | 19 | 74 | 183 | 1 | 97 | 97 | 80 | 82 | 83 | 84 | 86 | 86 | 88 | 95 | 98 | 79 | 80 | 82 | 88 | 92 | 94 | 88 | 92 | 92 |
|  | 1 | 20 | 67 | 171 | 1 | 90 | 89 | 75 | 74 | 75 | 82 | 88 | 97 | 98 | 102 | 100 | 80 | 82 | 83 | 76 | 77 | 80 | 105 | 100 | 105 |
|  | 1 | 19 | 87 | 174 | 1 | 96 | 96 | 80 | 80 | 80 | 82 | 90 | 90 | 90 | 92 | 91 | 80 | 81 | 82 | 86 | 88 | 92 | 98 | 102 | 99 |
|  | 1 | 17 | 64 | 176 | 1 | 89 | 90 | 72 | 71 | 72 | 88 | 92 | 90 | 100 | 98 | 99 | 70 | 72 | 73 | 88 | 91 | 90 | 100 | 98 | 97 |
|  | 1 | 31 | 80 | 181 | 1 | 94 | 95 | 80 | 78 | 80 | 90 | 98 | 92 | 105 | 108 | 110 | 79 | 78 | 80 | 90 | 93 | 92 | 108 | 110 | 110 |
|  | 1 | 17 | 77 | 181 | 1 | 98 | 98 | 82 | 83 | 82 | 100 | 105 | 105 | 105 | 107 | 109 | 81 | 83 | 80 | 106 | 110 | 109 | 120 | 122 | 125 |
|  | 1 | 26 | 70 | 174 | 1 | 90 | 90 | 72 | 72 | 75 | 98 | 100 | 100 | 105 | 105 | 110 | 63 | 65 | 67 | 97 | 100 | 105 | 110 | 110 | 115 |
|  | 1 | 18 | 70 | 178 | 1 | 90 | 90 | 70 | 71 | 72 | 84 | 80 | 81 | 90 | 90 | 90 | 69 | 70 | 71 | 80 | 85 | 87 | 91 | 90 | 87 |
|  | 1 | 33 | 70 | 172 | 1 | 93 | 93 | 72 | 70 | 72 | 78 | 80 | 80 | 80 | 80 | 80 | 67 | 69 | 70 | 88 | 90 | 90 | 87 | 90 | 90 |
|  | 1 | 18 | 80 | 191 | 1 | 100 | 100 | 82 | 81 | 83 | 101 | 105 | 105 | 100 | 102 | 107 | 74 | 75 | 77 | 101 | 105 | 105 | 106 | 104 | 100 |
|  | 1 | 20 | 74 | 187 | 1 | 100 | 100 | 85 | 87 | 88 | 85 | 90 | 91 | 95 | 97 | 100 | 73 | 75 | 76 | 88 | 90 | 92 | 102 | 100 | 97 |
|  | 1 | 18 | 70 | 172 | 1 | 89 | 89 | 68 | 68 | 69 | 86 | 87 | 88 | 87 | 89 | 90 | 67 | 71 | 70 | 87 | 90 | 88 | 96 | 97 | 100 |
|  | 1 | 21 | 70 | 181 | 1 | 94 | 94 | 72 | 74 | 74 | 95 | 100 | 100 | 106 | 110 | 110 | 67 | 69 | 70 | 97 | 100 | 102 | 110 | 114 | 112 |
|  | 1 | 23 | 67 | 178 | 1 | 93 | 93 | 72 | 73 | 72 | 85 | 83 | 85 | 85 | 86 | 90 | 62 | 64 | 65 | 89 | 88 | 90 | 83 | 84 | 85 |
|  | 1 | 27 | 71 | 179 | 1 | 92 | 92 | 74 | 75 | 76 | 88 | 90 | 90 | 98 | 95 | 100 | 72 | 74 | 75 | 90 | 88 | 92 | 102 | 105 | 110 |
|  | 1 | 20 | 68 | 171 | 1 | 86 | 86 | 70 | 70 | 72 | 82 | 80 | 80 | 80 | 80 | 80 | 71 | 73 | 74 | 81 | 79 | 80 | 78 | 80 | 82 |
|  | 1 | 18 | 79 | 181 | 1 | 94 | 94 | 82 | 82 | 83 | 87 | 90 | 90 | 100 | 103 | 107 | 83 | 85 | 86 | 90 | 93 | 92 | 105 | 110 | 110 |
|  | 1 | 25 | 70 | 182 | 1 | 92 | 92 | 72 | 72 | 72 | 78 | 80 | 80 | 87 | 90 | 92 | 72 | 71 | 70 | 86 | 88 | 89 | 90 | 93 | 95 |
|  | 1 | 18 | 75 | 191 | 1 | 98 | 98 | 80 | 81 | 83 | 85 | 87 | 90 | 110 | 110 | 110 | 73 | 75 | 76 | 90 | 92 | 90 | 100 | 102 | 104 |
|  | 1 | 17 | 65 | 176 | 1 | 87 | 87 | 70 | 68 | 70 | 85 | 85 | 88 | 87 | 90 | 90 | 67 | 69 | 70 | 83 | 82 | 85 | 88 | 90 | 94 |
|  | 1 | 19 | 51 | 166 | 1 | 85 | 85 | 70 | 70 | 70 | 70 | 80 | 80 | 80 | 80 | 81 | 65 | 68 | 70 | 78 | 80 | 82 | 85 | 80 | 82 |
|  | 1 | 28 | 70 | 175 | 1 | 89 | 89 | 70 | 68 | 68 | 80 | 80 | 80 | 90 | 90 | 90 | 67 | 69 | 70 | 79 | 80 | 83 | 95 | 94 | 90 |
|  | 1 | 25 | 81 | 185 | 1 | 98 | 98 | 80 | 80 | 80 | 105 | 100 | 102 | 105 | 110 | 110 | 77 | 79 | 80 | 100 | 104 | 102 | 112 | 115 | 110 |
|  | 1 | 32 | 70 | 180 | 1 | 91 | 91 | 70 | 69 | 70 | 90 | 95 | 95 | 115 | 115 | 115 | 67 | 69 | 69 | 102 | 106 | 104 | 119 | 120 | 117 |
|  | 1 | 19 | 75 | 176 | 1 | 90 | 89 | 76 | 75 | 76 | 89 | 89 | 90 | 98 | 100 | 97 | 75 | 78 | 77 | 88 | 90 | 92 | 96 | 98 | 99 |
|  | 1 | 18 | 86 | 195 | 1 | 97 | 98 | 80 | 81 | 82 | 98 | 100 | 100 | 114 | 120 | 120 | 79 | 77 | 81 | 108 | 110 | 111 | 113 | 115 | 115 |
|  | 1 | 19 | 67 | 191 | 1 | 100 | 100 | 82 | 82 | 83 | 98 | 98 | 100 | 105 | 108 | 110 | 80 | 82 | 83 | 102 | 105 | 104 | 106 | 113 | 110 |
|  | 1 | 20 | 64 | 169 | 1 | 86 | 86 | 68 | 68 | 68 | 86 | 87 | 86 | 80 | 95 | 89 | 66 | 69 | 68 | 86 | 86 | 90 | 89 | 92 | 94 |
|  | 1 | 23 | 75 | 179 | 1 | 93 | 93 | 72 | 73 | 74 | 85 | 86 | 87 | 99 | 95 | 100 | 67 | 69 | 70 | 80 | 82 | 80 | 97 | 100 | 100 |
|  | 1 | 19 | 65 | 170 | 1 | 89 | 89 | 71 | 72 | 70 | 100 | 98 | 100 | 100 | 100 | 97 | 70 | 69 | 73 | 90 | 94 | 92 | 97 | 100 | 105 |
|  | 1 | 31 | 76 | 186 | 1 | 97 | 97 | 78 | 78 | 80 | 95 | 99 | 94 | 103 | 105 | 108 | 78 | 80 | 81 | 97 | 98 | 103 | 105 | 110 | 108 |
|  | 1 | 21 | 70 | 181 | 1 | 95 | 95 | 76 | 77 | 76 | 93 | 93 | 92 | 108 | 100 | 105 | 68 | 69 | 70 | 100 | 97 | 96 | 107 | 108 | 104 |
|  | 1 | 26 | 73 | 173 | 1 | 89 | 89 | 68 | 68 | 70 | 94 | 95 | 95 | 103 | 107 | 112 | 67 | 68 | 70 | 105 | 108 | 107 | 108 | 109 | 112 |
|  | 1 | 26 | 71 | 174 | 1 | 89 | 89 | 70 | 69 | 68 | 104 | 105 | 105 | 103 | 108 | 102 | 63 | 66 | 65 | 95 | 96 | 98 | 110 | 108 | 101 |
|  | 1 | 32 | 72 | 180 | 1 | 91 | 91 | 70 | 70 | 70 | 90 | 90 | 92 | 90 | 90 | 95 | 66 | 68 | 69 | 94 | 95 | 100 | 90 | 89 | 96 |
|  | 1 | 28 | 75 | 181 | 1 | 96 | 96 | 76 | 78 | 78 | 102 | 100 | 100 | 100 | 101 | 100 | 75 | 74 | 77 | 102 | 104 | 100 | 97 | 104 | 106 |
|  | 1 | 20 | 90 | 195 | 1 | 101 | 100 | 82 | 82 | 82 | 98 | 100 | 98 | 115 | 112 | 117 | 76 | 79 | 77 | 95 | 96 | 98 | 100 | 111 | 108 |
|  | 1 | 18 | 88 | 206 | 2 | 104 | 104 | 86 | 87 | 88 | 104 | 104 | 105 | 106 | 107 | 110 | 72 | 74 | 76 | 100 | 105 | 103 | 103 | 105 | 106 |
|  | 1 | 18 | 76 | 191 | 2 | 99 | 99 | 78 | 81 | 80 | 83 | 83 | 85 | 87 | 90 | 90 | 75 | 75 | 75 | 89 | 90 | 92 | 82 | 90 | 96 |
|  | 1 | 24 | 97 | 191 | 2 | 101 | 101 | 85 | 84 | 85 | 90 | 90 | 90 | 98 | 100 | 103 | 78 | 80 | 81 | 90 | 88 | 91 | 90 | 100 | 100 |
|  | 1 | 18 | 93 | 198 | 2 | 109 | 109 | 88 | 88 | 88 | 100 | 100 | 100 | 105 | 105 | 110 | 82 | 85 | 85 | 94 | 95 | 97 | 110 | 111 | 115 |
|  | 1 | 29 | 86 | 194 | 2 | 103 | 103 | 84 | 85 | 84 | 105 | 110 | 105 | 115 | 115 | 110 | 73 | 75 | 75 | 107 | 110 | 112 | 107 | 110 | 110 |
|  | 1 | 24 | 99 | 210 | 2 | 116 | 116 | 92 | 92 | 95 | 100 | 102 | 102 | 115 | 110 | 115 | 87 | 90 | 91 | 98 | 102 | 100 | 108 | 110 | 112 |
|  | 1 | 33 | 107 | 205 | 2 | 107 | 107 | 90 | 91 | 90 | 100 | 100 | 105 | 105 | 110 | 107 | 87 | 88 | 90 | 100 | 105 | 102 | 105 | 110 | 110 |
|  | 1 | 25 | 79 | 196 | 2 | 104 | 104 | 89 | 90 | 90 | 120 | 121 | 120 | 122 | 121 | 125 | 73 | 75 | 77 | 120 | 119 | 118 | 121 | 119 | 123 |
|  | 1 | 32 | 105 | 205 | 2 | 107 | 107 | 90 | 90 | 91 | 100 | 100 | 100 | 115 | 117 | 112 | 83 | 85 | 86 | 107 | 105 | 108 | 114 | 112 | 115 |
|  | 1 | 23 | 104 | 210 | 2 | 116 | 116 | 96 | 96 | 97 | 98 | 98 | 100 | 119 | 115 | 120 | 82 | 85 | 86 | 98 | 100 | 102 | 110 | 115 | 117 |
|  | 1 | 25 | 83 | 178 | 2 | 95 | 95 | 72 | 72 | 72 | 87 | 88 | 91 | 94 | 91 | 91 | 67 | 69 | 70 | 84 | 80 | 82 | 98 | 100 | 105 |
|  | 1 | 18 | 76 | 190 | 2 | 99 | 99 | 80 | 82 | 82 | 83 | 80 | 81 | 96 | 95 | 98 | 68 | 75 | 80 | 80 | 82 | 79 | 98 | 95 | 95 |
|  | 1 | 29 | 89 | 195 | 2 | 103 | 103 | 86 | 86 | 85 | 108 | 110 | 110 | 112 | 110 | 110 | 72 | 73 | 76 | 105 | 110 | 108 | 116 | 112 | 115 |
|  | 1 | 32 | 77 | 186 | 2 | 98 | 97 | 75 | 74 | 73 | 102 | 104 | 105 | 105 | 110 | 106 | 73 | 75 | 72 | 100 | 105 | 104 | 111 | 115 | 112 |
|  | 1 | 18 | 93 | 198 | 2 | 109 | 109 | 88 | 89 | 89 | 92 | 95 | 90 | 104 | 106 | 107 | 73 | 77 | 78 | 87 | 85 | 88 | 99 | 102 | 100 |
|  | 2 | 18 | 82 | 180 | 3 | 93 | 93 | 72 | 74 | 74 | 89 | 90 | 92 | 90 | 92 | 95 | 68 | 70 | 71 | 80 | 78 | 82 | 96 | 94 | 95 |
|  | 2 | 18 | 58 | 178 | 3 | 91 | 91 | 76 | 74 | 74 | 87 | 85 | 90 | 105 | 107 | 110 | 74 | 75 | 76 | 82 | 87 | 88 | 106 | 108 | 109 |
|  | 2 | 18 | 67 | 185 | 3 | 98 | 97 | 82 | 80 | 80 | 94 | 94 | 92 | 97 | 98 | 100 | 82 | 81 | 85 | 95 | 97 | 100 | 99 | 100 | 102 |
|  | 2 | 22 | 69 | 183 | 3 | 93 | 93 | 76 | 77 | 78 | 95 | 95 | 96 | 90 | 92 | 92 | 78 | 80 | 81 | 94 | 95 | 98 | 110 | 108 | 111 |
|  | 2 | 24 | 67 | 187 | 3 | 100 | 100 | 82 | 83 | 83 | 90 | 90 | 91 | 90 | 90 | 92 | 85 | 83 | 84 | 95 | 94 | 92 | 105 | 108 | 110 |
|  | 2 | 21 | 60 | 179 | 3 | 90 | 90 | 76 | 76 | 76 | 68 | 70 | 70 | 83 | 85 | 85 | 68 | 69 | 70 | 74 | 76 | 78 | 90 | 92 | 95 |
|  | 2 | 18 | 67 | 180 | 3 | 93 | 93 | 78 | 78 | 78 | 88 | 88 | 88 | 90 | 89 | 90 | 82 | 84 | 85 | 77 | 78 | 80 | 94 | 98 | 95 |
|  | 2 | 18 | 61 | 186 | 3 | 95 | 95 | 78 | 77 | 79 | 94 | 94 | 95 | 95 | 96 | 95 | 77 | 78 | 80 | 100 | 102 | 98 | 104 | 105 | 108 |
|  | 2 | 18 | 64 | 183 | 3 | 94 | 94 | 77 | 76 | 78 | 94 | 95 | 95 | 100 | 100 | 100 | 76 | 78 | 79 | 94 | 96 | 99 | 98 | 99 | 102 |
|  | 2 | 27 | 68 | 179 | 3 | 94 | 94 | 76 | 76 | 75 | 92 | 93 | 92 | 98 | 100 | 100 | 80 | 82 | 80 | 95 | 94 | 92 | 95 | 98 | 100 |
|  | 2 | 29 | 78 | 189 | 3 | 101 | 102 | 86 | 85 | 86 | 100 | 102 | 100 | 115 | 118 | 115 | 85 | 86 | 87 | 112 | 110 | 116 | 115 | 118 | 120 |
|  | 2 | 24 | 58 | 167 | 3 | 83 | 83 | 68 | 69 | 70 | 92 | 93 | 94 | 98 | 100 | 99 | 72 | 75 | 74 | 88 | 90 | 91 | 105 | 110 | 108 |
|  | 2 | 18 | 55 | 170 | 3 | 84 | 84 | 68 | 69 | 68 | 90 | 88 | 90 | 90 | 90 | 90 | 70 | 69 | 72 | 82 | 80 | 80 | 90 | 96 | 98 |
|  | 2 | 18 | 78 | 187 | 3 | 89 | 89 | 70 | 72 | 71 | 92 | 94 | 94 | 89 | 90 | 90 | 78 | 80 | 77 | 90 | 95 | 97 | 80 | 85 | 82 |
|  | 2 | 18 | 75 | 180 | 3 | 85 | 85 | 70 | 70 | 70 | 80 | 80 | 80 | 85 | 86 | 88 | 72 | 73 | 75 | 78 | 80 | 90 | 90 | 95 | 105 |
|  | 2 | 24 | 58 | 166 | 3 | 83 | 83 | 67 | 67 | 68 | 94 | 95 | 95 | 100 | 100 | 100 | 75 | 74 | 74 | 105 | 109 | 110 | 96 | 98 | 100 |
|  | 2 | 27 | 68 | 179 | 3 | 94 | 94 | 78 | 78 | 78 | 95 | 95 | 94 | 100 | 100 | 100 | 77 | 78 | 80 | 100 | 97 | 102 | 100 | 102 | 100 |
|  | 2 | 18 | 61 | 186 | 3 | 95 | 95 | 80 | 80 | 82 | 80 | 90 | 100 | 103 | 105 | 105 | 78 | 81 | 80 | 90 | 91 | 92 | 100 | 104 | 102 |
|  | 2 | 18 | 64 | 184 | 3 | 98 | 97 | 82 | 82 | 80 | 88 | 90 | 90 | 100 | 100 | 98 | 85 | 87 | 88 | 98 | 100 | 98 | 105 | 100 | 102 |
|  | 2 | 18 | 65 | 170 | 3 | 81 | 81 | 66 | 67 | 66 | 82 | 82 | 80 | 90 | 90 | 92 | 73 | 72 | 75 | 90 | 88 | 90 | 95 | 98 | 100 |
|  | 2 | 28 | 70 | 185 | 3 | 95 | 95 | 72 | 72 | 72 | 90 | 92 | 90 | 100 | 100 | 98 | 71 | 68 | 70 | 100 | 98 | 97 | 102 | 100 | 98 |
|  | 2 | 18 | 58 | 175 | 3 | 85 | 85 | 72 | 71 | 72 | 85 | 80 | 85 | 90 | 92 | 90 | 78 | 79 | 80 | 90 | 93 | 96 | 92 | 94 | 95 |
|  | 2 | 20 | 60 | 177 | 3 | 85 | 85 | 70 | 70 | 70 | 98 | 98 | 100 | 100 | 102 | 100 | 69 | 71 | 72 | 90 | 94 | 93 | 98 | 103 | 104 |
|  | 2 | 18 | 60 | 168 | 3 | 80 | 80 | 65 | 65 | 64 | 94 | 94 | 95 | 94 | 95 | 95 | 68 | 69 | 70 | 90 | 93 | 94 | 92 | 97 | 99 |
|  | 2 | 21 | 69 | 181 | 3 | 90 | 90 | 76 | 75 | 76 | 100 | 100 | 100 | 93 | 95 | 95 | 81 | 82 | 81 | 98 | 100 | 95 | 95 | 92 | 91 |
|  | 2 | 21 | 75 | 179 | 3 | 88 | 88 | 72 | 72 | 71 | 85 | 82 | 84 | 88 | 90 | 90 | 69 | 73 | 70 | 87 | 90 | 92 | 96 | 91 | 90 |
|  | 2 | 20 | 55 | 172 | 3 | 83 | 83 | 70 | 70 | 70 | 81 | 82 | 80 | 84 | 84 | 82 | 68 | 70 | 70 | 81 | 83 | 80 | 91 | 90 | 94 |
|  | 2 | 20 | 68 | 182 | 3 | 95 | 95 | 80 | 80 | 82 | 100 | 102 | 100 | 108 | 108 | 110 | 92 | 91 | 90 | 103 | 104 | 106 | 108 | 110 | 112 |
|  | 2 | 27 | 62 | 176 | 3 | 89 | 89 | 68 | 68 | 69 | 90 | 92 | 93 | 98 | 100 | 100 | 76 | 78 | 77 | 88 | 92 | 93 | 100 | 105 | 108 |
|  | 2 | 18 | 77 | 182 | 3 | 94 | 94 | 78 | 78 | 80 | 75 | 78 | 75 | 84 | 82 | 82 | 75 | 74 | 77 | 80 | 78 | 82 | 88 | 85 | 92 |
|  | 2 | 27 | 103 | 193 | 3 | 100 | 100 | 82 | 84 | 84 | 80 | 78 | 80 | 93 | 94 | 94 | 79 | 81 | 82 | 82 | 80 | 84 | 94 | 95 | 92 |
|  | 2 | 24 | 90 | 185 | 3 | 98 | 98 | 80 | 80 | 80 | 84 | 82 | 83 | 84 | 85 | 86 | 77 | 79 | 80 | 80 | 78 | 82 | 86 | 88 | 88 |
|  | 2 | 18 | 60 | 173 | 3 | 90 | 90 | 75 | 76 | 77 | 88 | 85 | 88 | 92 | 96 | 95 | 79 | 81 | 81 | 82 | 90 | 87 | 100 | 98 | 103 |
|  | 2 | 24 | 78 | 180 | 3 | 93 | 93 | 77 | 76 | 78 | 100 | 98 | 98 | 115 | 115 | 115 | 81 | 79 | 80 | 97 | 98 | 95 | 114 | 112 | 110 |
